# Methylsulfonylmethane Increases the Alveolar Bone Density of Mandibles in Aging Female Mice

**DOI:** 10.3389/fphys.2021.708905

**Published:** 2021-10-04

**Authors:** Hanan Aljohani, Linda T. Senbanjo, Mohammed Al Qranei, Joseph P. Stains, Meenakshi A. Chellaiah

**Affiliations:** ^1^Department of Oncology and Diagnostic Sciences, School of Dentistry, University of Maryland, Baltimore, Baltimore, MD, United States; ^2^Department of Oral Medicine and Diagnostics Sciences, School of Dentistry, King Saud University, Riyadh, Saudi Arabia; ^3^Department of Preventive Dental Sciences, School of Dentistry, Imam Abdulrahman Bin Faisal University, Dammam, Saudi Arabia; ^4^Department of Orthopedics, University of Maryland School of Medicine, Baltimore, MD, United States

**Keywords:** osteoblasts, aging mice, bone formation, osteoclasts, methylsulfonylmethane

## Abstract

Methylsulfonylmethane (MSM) is a naturally occurring anti-inflammatory compound that effectively treats multiple degenerative diseases such as osteoarthritis and acute pancreatitis. Our previous studies have demonstrated the ability of MSM to differentiate stem cells from human exfoliated deciduous (SHED) teeth into osteoblast-like cells. This study examined the systemic effect of MSM in 36-week-old aging C57BL/6 female mice *in vivo* by injecting MSM for 13 weeks. Serum analyses showed an increase in expression levels of bone formation markers [osteocalcin (OCN) and procollagen type 1 intact N-terminal propeptide (P1NP)] and a reduction in bone resorption markers [tartrate-resistant acid phosphatase (TRAP) and C-terminal telopeptide of type I collag (CTX-I)] in MSM-injected animals. Micro-computed tomographic images demonstrated an increase in trabecular bone density in mandibles. The trabecular bone density tended to be higher in the femur, although the increase was not significantly different between the MSM- and phosphate-buffered saline (PBS)-injected mice. In mandibles, an increase in bone density with a corresponding decrease in the marrow cavity was observed in the MSM-injected mice. Furthermore, immunohistochemical analyses of the mandibles for the osteoblast-specific marker – OCN, and the mesenchymal stem cell-specific marker – CD105 showed a significant increase and decrease in OCN and CD105 positive cells, respectively. Areas of bone loss were observed in the inter-radicular region of mandibles in control mice. However, this loss was considerably decreased due to stimulation of bone formation in response to MSM injection. In conclusion, our study has demonstrated the ability of MSM to induce osteoblast formation and function *in vivo*, resulting in increased bone formation in the mandible. Hence, the application of MSM and stem cells of interest may be the right combination in alveolar bone regeneration under periodontal or other related diseases that demonstrate bone loss.

## Introduction

Bone loss associated with osteoporosis represents a significant health care problem, and it is related to increased activation of osteoclast bone resorption function (Mundy, 2007; Khosla et al., 2012; Soysa and Alles, 2016; Cai et al., 2017). Many contributing factors can cause osteoporosis, and one such aspect is aging (Demontiero et al., 2012). Increased pro-inflammatory markers in older adults represent aging-related osteoporosis (Weitzmann and Pacifici, 2006; Pacifici, 2008). Increased inflammation in aged mammals is correlated with higher circulating pro-inflammatory cytokines than young adults (Abdelmagid et al., 2015). The reason is that increased circulating pro-inflammatory mediators could induce molecular changes in the periodontal tissue and exaggerate bone loss in older adults (Liang et al., 2010). In addition, tooth loss in older adults is linked with periodontal disease (Koduganti et al., 2009).

Experiments with animals and studies with humans have implicated pro-inflammatory cytokines (e.g., interleukin-1, tumor necrosis factor-alpha, and interleukin-6) as primary mediators of physiologic and pathological bone remodeling (Goldring, 2003). Chronic inflammation in aging is specified by increased inflammatory mediators, osteoclast activation, and bone loss. Furthermore, aging leads to underlying modifications in the differentiation of mesenchymal stem cells (MSCs) and therefore impaired osteoblast differentiation and bone formation (Abdelmagid et al., 2015). The deregulation of the balance between bone formation and bone resorption causes age-related osteoporosis. The deregulation is related to increase osteoclast formation and bone resorption and decrease osteoblast differentiation and bone formation. Appropriate alteration of the inflammatory condition is required for typical bone remodeling. Therefore, it is essential to identify a new anti-inflammatory agent to increase osteoblast function and bone formation.

Methylsulfonylmethane (MSM) is a naturally occurring organosulfur compound with several health benefits. It is a potent anti-inflammatory compound, which reduces chronic inflammation and relieves pain. It is used as a dietary supplement with glucosamine and chondroitin sulfate to treat arthritis (Usha and Naidu, 2004; Kim et al., 2006; Gregory et al., 2008; Lubis et al., 2017). An increase in pro-inflammatory cytokines (e.g., IL-6 and TNF-α) has been observed due to activation of the transcriptional factor NF-κB. It is worth noting that MSM reduced the expression of these cytokines by inhibiting NF-κB activity (Kim et al., 2009; Ahn et al., 2015). Moreover, MSM is a selective inhibitor of the NLRP3 inflammasome activation in human macrophages *in vitro*; analyses in mice corroborated this observation *in vivo* (Ahn et al., 2015).

Studies by others and we elucidated MSM’s effect on bone formation using stem cells such as MSCs, human periodontal ligament stem cells (hPDLSCs), and stem cells from human exfoliated deciduous teeth (SHED) (Joung et al., 2012; Aljohani et al., 2019; Ha and Choung, 2020). MSM induces osteoblast differentiation *via* activating the JAK2/STAT5b pathway in MSCs (Joung et al., 2012). We found that MSM significantly increases transglutaminase-2 (TG-2) activity and its interaction with extracellular matrix (ECM) proteins such as collagen type 1 and osteopontin (Aljohani et al., 2019). An increase in the expression of osteogenic markers and mineralization by MSM in PDLSCs and SHED suggests that MSM is suitable not only for the inhibition of inflammatory-related events (Kim et al., 2009; Ahn et al., 2015) and diseases but also for increasing bone formation (Joung et al., 2012; Aljohani et al., 2019; Ha and Choung, 2020). *In vivo* analysis with hPDLSCs in calvarial defect and transplantation models indicate that MSM could be used with stem cells for bone regeneration *in vivo* (Ha and Choung, 2020). Mice naturally develop accelerated periodontal bone loss as a function of age (Liang et al., 2010). In addition, aging can cause bone loss in trabecular bone microarchitecture, leading to bone fracture and tooth loss (Huttner et al., 2009; Koduganti et al., 2009; Willinghamm et al., 2010; Eastell et al., 2016). Thus, we believed that the aging mouse model represents a genuinely chronic model to study possible periodontal tissue loss and restoration or remodeling mechanisms. Therefore, we proceeded to identify the effect of MSM on bone formation by osteoblasts in the aging mouse model. Here, we aim to relate the influence of MSM on the trabecular bone density of the femoral head to the mandible. Female C57BL/6 mice at 36 weeks of age were used for aging-related studies (Jilka, 2013). Histological and immunohistochemical analyses demonstrated that MSM could be an applicable osteogenic element in treating bone loss under inflammation, including aging and post-menopausal osteoporosis conditions.

## Materials and Methods

### Osteoblast Studies

#### Cell Culture

MC3T3-E1 (mouse mesenchyme stem cells) and UMR-106 (rat osteoblast-like cells) were obtained from American Type Culture Collection (ATCC, Manassas, VA, United States). SHED were a kind gift from Dr. Jacques Nör (University of Michigan, Ann Arbor, MI, United States). Briefly, SHED were collected from exfoliated deciduous incisors of 7- to 8-year-old children. Guidelines set and approved by the National Institutes of Health Office of Human Subjects Research were followed during the isolation procedure (Bento et al., 2013). Briefly, the pulp from a remnant crown was digested in a solution containing 3 mg/ml collagenase type I and 4 mg/ml dispase (Worthington Biochem, Freehold, NJ, United States and Roche Molecular Biochemicals, Pleasanton, CA, United States, respectively) for 1 h at 37°C. After digestion, the solution was passed through a 70-μm strainer (Falcon) to obtain a single-cell suspension as described (Gronthos et al., 2000).

UMR-106 cells were cultured in DMEM media containing 10% FBS, 1% penicillin/streptomycin, and 0.05% Gentamicin. In contrast, MC3T3-E1 cells and SHED were maintained in α-minimal essential medium (MEM) with 10% fetal bovine serum and 1% penicillin/streptomycin. All cells were maintained at 37°C in 5% CO_2_, and the media was changed every 3 days. For osteogenic differentiation, cells were incubated with osteogenic medium (OM), consisting of osteogenic factors, such as 50 μM ascorbic acid, 5 mM β-glycerophosphate, and 0.05% Gentamicin. In addition, some cultures were treated with MSM in the basal medium (BM) with no osteogenic factors.

#### Alkaline Phosphatase Activity Analysis

Alkaline phosphatase (ALP) activity was measured using the colorimetric assay (Aljohani et al., 2019). Cells were seeded in a six-well plate in MSM (20 mM) presence or absence for 7 days, and lysates were made as described (Aljohani et al., 2019). An equal amount of protein was used in triplicates in a 96-well plate to measure the activity. The absorbance was measured (405 nm) in a microplate reader (Cytation3 image) with integrated imaging software (Gen5 version 2.09) after the addition of *p*-nitrophenyl phosphate (10 μl; Sigma, St. Louis, MO, United States) to each well.

#### Alizarin Red S Staining and Von Kossa Staining

UMR-106 cells seeded and incubated for 7 days in a six-well plate in the presence and absence of MSM (20 mM) were used to determine the effect of MSM on matrix mineralization. Cells without any MSM but grown in the OM were used as controls. Alizarin red S (ARS) is used to stain cells after washing with phosphate-buffered saline (PBS) three times. Absolute ethanol was used to fix the cells for 30 min at room temperature. After ethanol aspiration, 2% ARS solution was added to each well and processed as described previously (Aljohani et al., 2019). For Von Kossa staining, cells were washed with PBS three times and fixed with 10% paraformaldehyde for 10 min at room temperature. After the aspiration of fixative and washing with PBS, a 5% silver nitrate solution was used as described previously (Aljohani et al., 2019). Scanning the culture plates stained for ARS and Von Kossa was done in the scanner (EPSON Perfection V200). Nikon Eclipse TE 2000-inverted light microscope were used to obtain magnified images (10× objective).

### Animals and Experimental Procedures

Thirty-six-week-old female C57BL6 mice weighing an average of 30 g were obtained from Charles River (MD, United States). Mice were maintained in the animal facility at the University of Maryland, Baltimore (School of Dentistry) animal care facility at room temperature (21 ± 1°C), with a 12 h light/12 h dark cycle. Pelleted mouse diet was fed *ad libitum*, and the mouse had free access to water. IACUC of the University of Maryland, Baltimore reviewed and approved the experimental procedures (approval number #417006, MD, United States). All experiments were performed under the relevant guidelines and regulations.

The mice were kept in the facility for a week for acclimatization before the injection. Mice were divided at random into two groups: a control group, injected with PBS (*n* = 6) as Group-1, and MSM injected mice (*n* = 6) as Group-2. Methylsulfonylmethane (PHR1346-1G, Sigma, St. Louis, MO, United States) was dissolved in PBS and injected subcutaneously (100 mg/kg) in a final volume of 100 μl. The injections were administered three times (i.e., alternate days) per week for 13 weeks. The animal weight was recorded every 4 weeks at the initial phase for 8 weeks and then after 3 and 2 weeks until the sacrifice time at 13 weeks. The mice were 49 weeks old at the time of sacrifice. Soft organs such as the heart, kidney, and liver have been isolated, and histological sections were prepared to assess any abnormalities caused by injections in these organs. Histological sections of these organs were stained with hematoxylin and eosin (H&E). Aperio ScanScope CS System (Vista, CA, United States) was used to scan the histological sections (bone and other tissues) (Aljohani et al., 2021). The assessment was performed blindly by a pathologist.

### Bone Histology and Histomorphometry Analysis

Bone histomorphometry analysis was performed as described previously (Chellaiah et al., 2003; Aljohani et al., 2021). The tibia and mandibles were stained with H&E and tartrate-resistant acid phosphatase (TRAP) staining according to the manufacturer’s protocols (Sigma, St. Louis, MO, United States). Stained sections were scanned and analyzed using the Aperio Scanscope CS instrument (Aperio Scanscope CS system, Vista, CA, United States). The number of TRAP-positive osteoclasts and cuboidal osteoblasts adherent to the bone surface were counted using the Fiji (ImageJ) software.

### Immunohistochemistry Analyses in Bone Sections

Immunohistochemistry was performed as described (Gupta et al., 2012). After blocking the sections with the blocking solution (2.5% BSA or horse serum in PBS) for 60 min, at 4°C, the slides were incubated overnight at 4°C with the primary antibody (Abcam, Cambridge, MA, United States) of interest [e.g., OCN (rabbit polyclonal), or CD105 (Mouse monoclonal)] which was diluted (1:100) in blocking solution. After washing with PBS, the sections were then incubated with the corresponding secondary antibodies for 60 min. The slides were then washed and developed as previously described (Gupta et al., 2012). Finally, immunostained sections were scanned using an Aperio Scanscope CS instrument (Aperio Scanscope CS system, Vista, CA, United States).

### Microcomputed Tomography Analysis

The femurs and mandibles were dissected from mice, and the soft tissues from the bones were removed. Bones were fixed in 4% paraformaldehyde for 2 days and then washed with PBS. Subsequently, bones were wrapped with gauze soaked in PBS and kept at 4°C. Three-dimensional microcomputed tomography (micro-CT) was performed on the femurs and mandibles (*n* = 6) using a Bruker Skyscan 1172 micro-CT scanner (Carteret, NJ, United States). Specimens were scanned with a 20 K resolution, 10 μm voxel size, 0.5 Al filter at 55 kV, and 167 μA, as described previously (Moorer et al., 2017). Bone morphology and microarchitecture were assessed at the distal femoral metaphysis in a region of interest (ROI) chosen for a range of 0.2–2.0 mm proximal to the distal femoral growth plate. For the mandibles, the ROI was selected in the inter-radicular area of the first molar. The skeletal parameters assessed by micro-CT followed published nomenclature guidelines (Dempster et al., 2013).

### Enzyme-Linked Immunosorbent Assay

Serum was separated from blood samples and frozen at −80°C until use. Serum markers of bone resorption (TRAP); (C-terminal telopeptide of type I collag, CTX-I) and of bone formation (osteocalcin, OCN); (procollagen type 1 intact N-terminal propeptide, P1NP), were measured in duplicate using enzyme-linked immunosorbent assay (ELISA) kits (Immunodiagnostics Systems, and LS-Bio Systems) according to the manufacturer’s instructions. In addition, serum calcium levels were also measured using a calcium detection kit (Biovision, Inc., Milpitas, CA, United States).

### Osteoclast Studies

#### Differentiation of Osteoclasts From RAW 264.7 Macrophage-Like Cell Line

Recombinant GST-RANKL was purified as described previously (Ma et al., 2010). Osteoclasts were generated from RAW 264.7 (ATCC^®^ TIB-71^TM^) cells as described (AlQranei et al., 2020). Mature multinucleated osteoclasts were observed from day three onward and used for various analyses.

#### Tartrate-Resistant Acid Phosphatase-Staining

For TRAP staining, undifferentiated macrophages were gently removed with a cell stripper solution, and multinucleated osteoclasts attached to the culture plates were used for staining as described previously (AlQranei et al., 2020). In brief, osteoclasts were fixed with 4% paraformaldehyde and washed three times with PBS. TRAP staining was done using the Leukocyte Acid Phosphatase Kit as described in the manufacturer’s protocol (Sigma, St. Louis, MO, United States; 387-A). Stained cells were photographed, and the number of mature osteoclasts was measured using Cytation5 image reader with software (Gen5 version 2.09).

#### Dentine Resorption Assay

Dentine slices were processed as described previously (Chellaiah et al., 2000). After processing, dentine slices were incubated overnight at 37°C in a serum-free α-MEM medium. The next day, an osteoclast suspension containing 2 × 10^4^ cells was gently added to the dentine slices. After adherence for 2 h, the culture media were replaced with serum-containing α-MEM containing RANKL with or without MSM at different concentrations (20 and 40 mM). After incubation for 48 h, dentine slices were processed and stained with acid hematoxylin (Sigma, St. Louis, MO, United States) for 6 min and washed well with water. Images of resorption pits were captured using a Nikon Eclipse TE 2000 inverted light microscope using a 20× and 40× objective (24).

### Statistical Analysis

All data are presented as mean ± SEM. Student’s *t*-test or Mann–Whitney *U* test was used to determine the statistical significance (Graph Pad Software, Graph Pad Inc., San Diego, CA, United States). The *p*-value < 0.05 is considered statistically significant.

## Results

### Analysis of the Effect of Methylsulfonylmethane on Bone Mineralization *in vitro*

To demonstrate the effect of MSM on ALP activity, we used MC3T3-E1 and UMR-106 osteoblastic cell lines. Consistent with the observation shown in SHED (Aljohani et al., 2019), MSM increased ALP activity in these cells in the basal growth medium (BM) *in vitro*. This increase was equivalent to an OM containing osteogenic factors (Supplementary Figure 1).

### Effects of Subcutaneous Injection of Methylsulfonylmethane on Body Weight Measurements and Soft Organs

We subsequently sought to determine the effect of MSM on bone formation *in vivo* using an aging mouse model. The injection was performed for 13 weeks, as described in section “Materials and Methods.” During the injection period, the mice looked healthy and exhibited normal behavior. Some mice exhibited hair loss when they first arrived at our facility. However, after 6 weeks of injection with MSM, these mice displayed hair growth compared to the PBS-injected control group. At the end of the injection period of 13 weeks, the mice injected with PBS demonstrated hair loss patches (Supplementary Figure 2A, white arrows) compared to the MSM group. The hair loss was reduced, and hair growth was observed in MSM injected group (Supplementary Figure 2A). No severe abnormalities were observed in the mice injected with PBS or MSM at the end of the injection period. It was also observed that MSM did not affect body weight changes compared to the PBS-injected mice (Supplementary Figure 2B). In histological sections, all soft organs (heart, kidney, and liver) showed normal histology with no signs of damage, inflammation, or defects (Supplementary Figure 3).

### Depiction of the Region of Interest Chosen for Microcomputed Tomography Analyses in Femoral and Mandibular Bones

**FIGURE 1:**
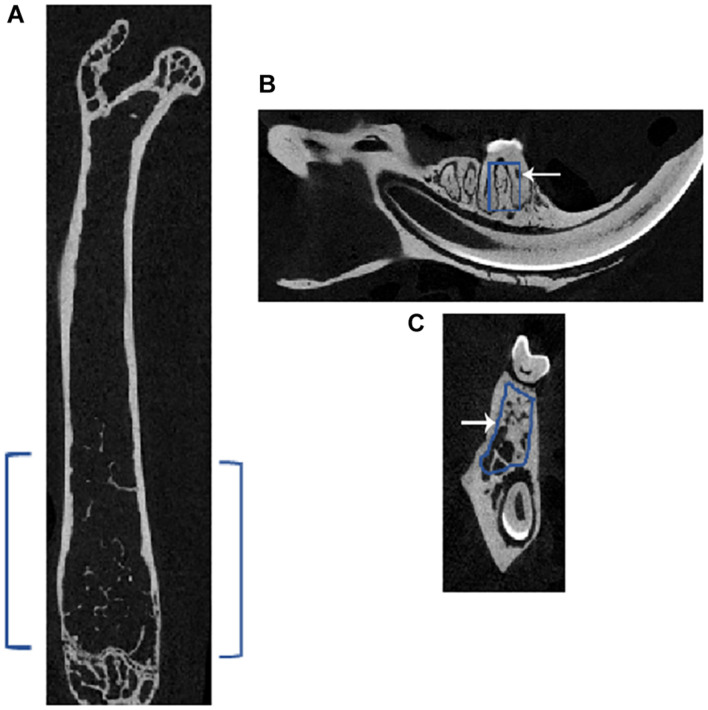
Representative micro-CT image of a femur and mandible of a mouse showing the region of interest (ROI) for subsequent analyses. Micro-CT of **(A)** femur in the axial plane showing the selected ROI in blue brackets, and mandible shows the chosen ROI, outlined in blue in the **(B)** sagittal and **(C)** coronal sections; white arrow points to the outline. In the femur, the ROI for the trabecular bone lies just below the growth plate region, which is called the metaphyseal area, where cancellous or trabecular bones are present. In the mandible, the ROI was chosen in the inter-radicular area between the roots of the first molar.

A two-dimensional (2D) model of the bones dissected from the injected mice was performed using the micro-CT. The ROI used in the femur and mandible is shown in Figure 1. In the femur, the chosen ROI for the trabecular bone lies below the growth plate region (i.e., in the metaphyseal area), where the cancellous or trabecular bones are present. In the mandible, the ROI was chosen in the inter-radicular area between the first molar roots. As depicted in Figure 1A, blue brackets in the femur and squares inside the sagittal (Figure 1B) and coronal (Figure 1C) sections indicate the ROI for scanning in micro-CT.

### Microcomputed Tomography Analyses in the Right Femurs of Mice Injected With Phosphate-Buffered Saline and Methylsulfonylmethane

**FIGURE 2:**
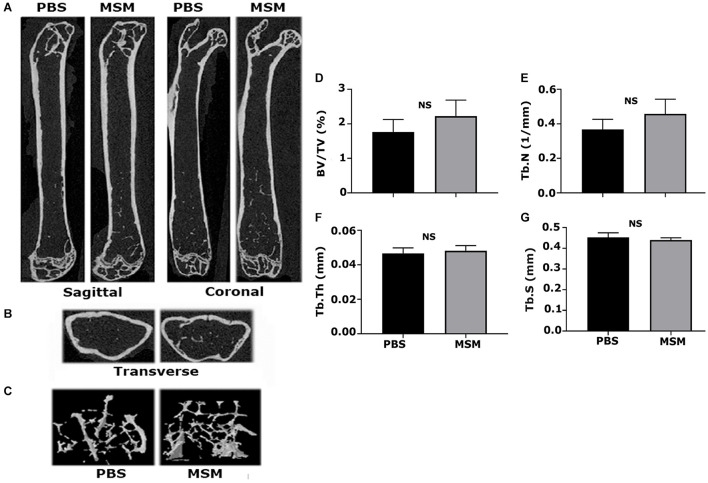
Microcomputed tomography analysis of trabecular and cortical bone isolated from mice femurs injected with PBS and MSM. **(A)** Representative longitudinal sections of the femur are shown in two different planes, i.e., the sagittal and coronal, **(B)** The ROI indicated in Figure 1 was used for transverse plane scanning, and **(C)** For three-dimensional (3D) construction. The micro-CT parameters were compared in six mice femurs injected with PBS and MSM. Statistical analyses were performed using the Mann–Whitney *U* test for morphometric parameters, including **(D)** bone volume to tissue volume (BV/TV), **(E)** trabecular number (Tb. N) and **(F)** trabecular thickness (Tb. Th), and **(G)** trabecular spacing (Tb. S), all shown as bar graphs. Data are shown as the mean ± SEM; NS, not statistically significant.

Microcomputed tomography images partially reveal the trabecular bone microarchitecture and cortical bone morphology (Figures 2A,B). The 3D reconstruction of the bone (Figure 2C) provides the visualization of trabecular bone density in the ROI chosen for scanning (Figure 1A). Although trabecular bone density appeared to be more in MSM-injected mice (Figure 2C; right panel) than PBS-injected controls, statistical analyses showed no significant changes in the trabecular number (Tb. N) (Figure 2E). Bone volume to tissue volume (BV/TV) tended to be higher in the MSM-treated mice than the control mice (Figures 2D–G), but the difference was not statistically significant. There also were no significant changes in the Tb. N, trabecular thickness (Tb. Th), and trabecular separation or spacing (Tb. S) in mice injected with MSM compared with PBS controls (Figures 2D–G).

### Microcomputed Tomography Analyses in the Mandible From Mice Injected With Phosphate-Buffered Saline and Methylsulfonylmethane

**FIGURE 3:**
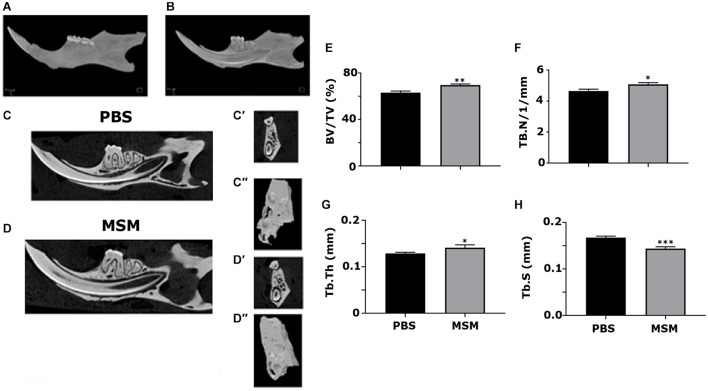
Microcomputed tomography analysis of mandibular bone in mice injected with PBS and MSM. **(A,B)** A 3D construction of the **(A)** whole mandible and a sagittal section displaying the **(B)** teeth inside their respective sockets are shown. Representative sagittal **(C,D)** and coronal **(C′,D′)** planes of mandibles isolated from mice injected with **(C)** PBS and **(D)** MSM are shown. For the micro-CT scanning of the panels **(C′,D′,C′′**, and **D′′)**, the ROI outlined (blue) in the **(B)** sagittal and **(C)** coronal planes are shown in Figure 1 were used. The micro-CT parameters were compared in six mice per group, and statistical analyses were performed using the Mann–Whitney *U* test for the indicated morphometric parameters **(E–H)**. The data are shown as mean ± SEM; ^∗^*p* < 0.05, ^∗∗^*p* < 0.01, ^∗∗∗^*p* < 0.001, vs. PBS-injected mice (BV/TV, bone volume-to-tissue volume; Tb. N, trabecular number; Tb, Th, trabecular thickness; Tb. S, trabecular spacing, or separation).

Microcomputed tomography scanning analyses of the alveolar bone of the mandibles showed a considerable increase in BV/TV, Tb. N, and Tb. Th accompanied by a decrease in the Tb. S in MSM-injected mice (Figure 3) compared with the PBS control mice. The ROI of the inter-radicular bone is projected with a blue rectangle in Figure 1C. Inter-radicular bones or septa are thin plates of bones that separate the roots of multi-rooted teeth. The 3D construction of inter-radicular bone is shown in Figures 3C″,D″. As can be seen, the bone density is higher in MSM-injected mice (Figure 3D″) compared to the control group. An increase was also observed in the inter-radicular bone of the mandible. These observations suggest that MSM can systematically induce bone formation, but its effect is more prominent in the bones in the mandibular region than in the long bones.

### Morphometric Analysis of the Tibial and Mandibular Bone Sections

**FIGURE 4:**
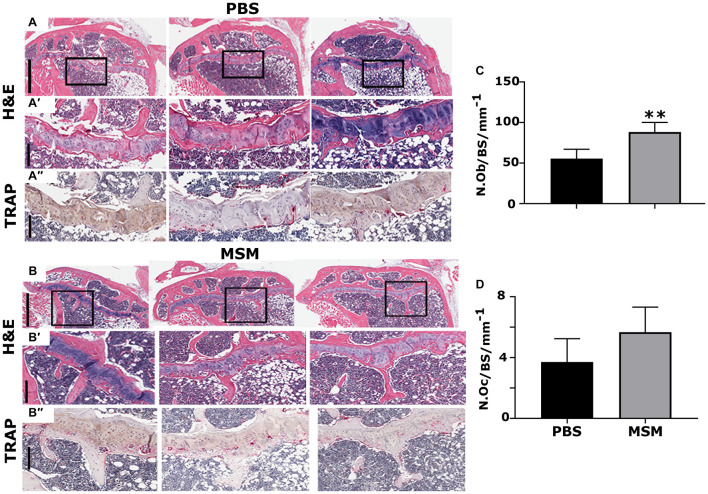
Histological assessment of the proximal right tibial bone sections in mice injected with PBS **(A)** and MSM **(B)** for 13 weeks. **(A,A′,B,B′)** Hematoxylin and Eosin (H&E) stained and **(A″,B″)** TRAP-stained proximal right tibial sections are shown in triplicate for each injection. Black squares in panels **(A,B)** indicate the region enlarged in panels **(A′,B′)**. The same area of the magnified bone section stained for TRAP is shown in panels **(A″,B″)**. Dark purple-stained cells seen attached to the bone surface are TRAP-positive osteoclasts [panels in **(A″,B″)**]. Scale bars represent 800 μm in panels **(A,B)**; 200 μm in the magnified H&E **(A′,B′)** and TRAP- **(A′,B′)** stained panels. Quantification of the number of osteoblasts (N. Ob) and osteoclasts (N. Oc) was performed in the tibial metaphyseal bone surface (BS) region. Quantification was performed using the Fiji (ImageJ) software, and the results are shown as the mean ± SEM of six mice per injection. A standard Student’s *t*-test was used to analyze *p*-values. ^∗∗^*p* < 0.01.

Methylsulfonylmethane injection appears to have increased the Tb. N in MSM-injected mice (Figures 4B,B′,B″) compared with PBS injected mice (Figures 4A,A′,A″). As detected by the micro-CT morphometry analyses, static histomorphometric measurements exhibited no significant femoral Tb. N or density changes in mice injected with MSM (Figure 2) compared to the control mice. Although no significant differences were noted in the number of the trabecular bone or its density, a considerable increase in the number of osteoblasts with no changes in the number of osteoclasts was observed between the two groups (Figures 4C,D).

**FIGURE 5:**
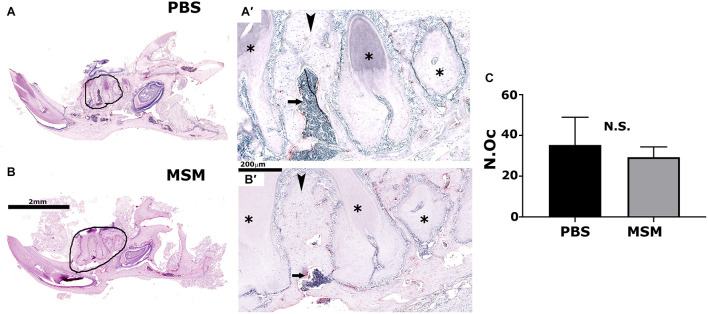
Tartrate-resistant acid phosphatase-stained mandible bone sections in mice injected with PBS **(A)** and MSM **(B)**. Representative TRAP-stained mandible sections for each injection are shown. Black circles in panels **(A,B)** indicate an enlarged view of the bone section in panels **(A′,B′)**. Arrowheads and arrows point to the inter-radicular bone and the bone marrow cavity, respectively, in PBS- and MSM-injected mice. Asterisks (^∗^) denote the dentine of adjacent teeth roots. Scale bars: 2 mm in panels **(A,B)** and 200 μm in panels **(A′,B′)**. Quantification of the number of osteoclasts (N.Oc) **(C)** was performed in six mice per group using Fiji software (ImageJ). A standard Student’s *t*-test was used to analyze the data. The number of osteoclasts was not significantly (NS) different between the experimental groups tested.

As seen in the micro-CT analyses (Figure 3D″), TRAP-stained mandibular bone sections of MSM-injected mice also exhibited an increase in bone density and a decrease in the marrow cavity (Figure 5B′, arrow) as compared with PBS-injected mice. Although the marrow cavity is wider in PBS-injected mice (Figure 5A′, arrow), the osteoclast number is not significantly different in mice injected with PBS or MSM (Figure 5C). Thus, an increase in the marrow cavity in PBS-injected mice may result from increased osteoclast activity and decreased or normal osteoblast function. While these findings support the prominent effect of MSM in bone formation, further studies are needed to confirm the exact mechanism by which MSM exerts its osteoinductive effects. Furthermore, *in vitro* experiments with osteoclasts derived from RAW cells demonstrated that MSM did not affect osteoclast differentiation or function *in vitro* (Supplementary Figure 4).

### Immunohistochemistry Analyses With Osteocalcin and CD105 Antibody

**FIGURE 6:**
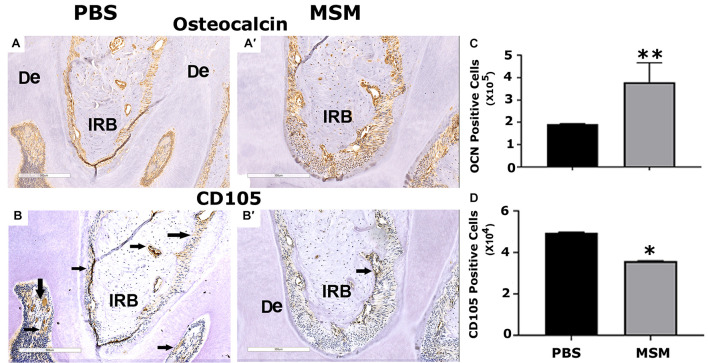
Immunohistochemistry analysis in mandible sections. Bone sections were stained with an antibody against **(A,B)** OCN or **(A′,B′)** CD105. Inter-radicular bone and dentine are abbreviated as IRB and De; arrows point to cells stained for CD105 **(A′,B′)**. Quantification of **(C)** OCN- and **(D)** CD105-positive cells was performed using Fiji (ImageJ) software, and the results are shown as the mean ± SEM of six mice per injection. A standard Student’s *t*-test was used to analyze *p*-values. ^∗∗^*p* < 0.01 and ^∗^*p* < 0.05. Scale bar: 300 μm.

We then used the mandible sections for immunohistochemistry analyses with OCN, a biomarker for osteoblast activity, and CD105, a stem cell marker. Immunostained sections demonstrated an increase in bone density in the inter-radicular bone region of the mandible with a significant rise in OCN-positive bone cells and a decline in CD105 positive stem cells in MSM injected mice (Figures 6,B,B′,C,D). In addition, as compared with PBS-injected mice (Figures 6A,A′), the inter-radicular bone width (IRB) is more in MSM injected mice (Figures 6B,B′). Thus, these observations indicate that MSM increases bone formation and the differentiation of CD105 positive stem cells into OCN-positive osteoblast-like cells.

### Analysis of Serum Biomarkers for Bone Formation and Resorption by Enzyme-Linked Immunosorbent Assay

**FIGURE 7:**
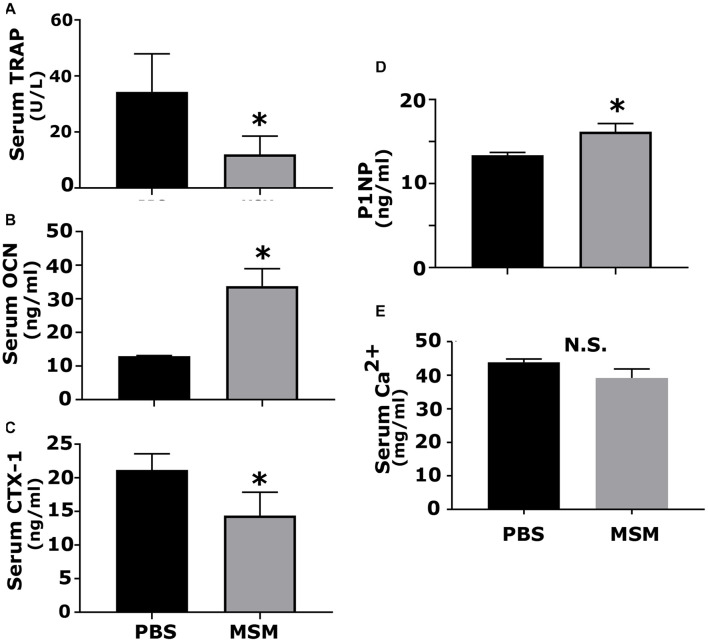
Analysis of the serum levels of indicated biomarkers of bone formation and resorption in mice injected with PBS and MSM. The serum levels of **(A)** TRAP, **(B)** OCN, **(C)** CTX-I, **(D)** P1NP, and **(E)** calcium were measured using ELISA kits. Serum from six mice per injection was used for the analyses, and each analysis was performed in triplicates. The results are presented as mean ± SEM; standard Student’s *t*-test was used to analyze the *p*-value; ^∗^*p* < 0.05; N.S., not statistically significant.

Serum was analyzed by ELISA for bone resorption (TRAP and CTX-I) and bone formation (OCN, P1NP) markers (Figure 7). An increase in osteoblast markers such as OCN and P1NP was observed in mice injected with MSM compared to PBS-injected mice (Figures 7B,D). Interestingly, we also detected a notable decrease in the levels of TRAP and CTX-I in the MSM-injected mice group as compared to the control group, which suggests that osteoclast activity may be reduced by MSM (Figures 7A,C) even though the osteoclast number was not significantly different between PBS- and MSM-injected mice (Figure 5C).

## Discussion

Our previous studies have shown that MSM influences the differentiation of SHED into osteoblast-like cells and their osteogenic potential. In SHED, TG-2 enzyme is involved in the cross-linking of ECM proteins (collagen and osteopontin) and the mineralization process *in vitro* in the presence of MSM (Aljohani et al., 2019). MC3T3 and UMR-106 cells are commonly used for *in vitro* studies. Validating the results of our earlier studies in SHED (Aljohani et al., 2019), here we showed that MSM increased ALP activity and mineralization in UMR-106 cells. Furthermore, besides MC3T3, SHED, and UMR-106 cells, MSM also increased the expression levels of osteogenic specific markers (ALP, osteopontin, OCN, RUNX2, and osterix) in hPDLSCs (Ha and Choung, 2020). We experimented with aging mice to comprehend whether *in vitro* findings of increased bone formation by MSM *in vitro* are also relevant *in vivo*. We performed a series of observations in aging mice, especially in the mandibular bone area, to elucidate whether MSM is an attractive therapeutic compound for the treatment of bone loss.

Bone loss occurs under conditions of periodontitis and osteoporosis, and both progress with increasing age (Jonasson and Rythen, 2016). Most clinical studies on the effects of human aging on periodontal tissues suggest a significant correlation between the aging and incidence of periodontal disease (Papapanou et al., 1989; Ismail et al., 1990; Haffajee et al., 1991; Huttner et al., 2009). MSM is commonly used as a supplement to treat arthritis and other inflammatory conditions (Butawan et al., 2017). Aging itself has been considered a chronic inflammatory state (Gibon et al., 2017). Periodontal tissues of aged mice have increased inflammation and elevated alveolar bone loss compared to young mice (Liang et al., 2010). Therefore, we used the aging mice model to identify the effect of MSM on bone cells. Mice were injected with MSM subcutaneously for 13 weeks. Our study demonstrated more bone loss in control mice which is diminished in MSM injected mice in the mandibular region.

We then did a series of studies in aging mice and analyzed the bone by micro-CT and histomorphometry analyses. We determined the quantitative differences in PBS- and MSM-injected mice by measuring BV/TV, Tb. Th, trabecular spacing, and Tb. N. Although bone loss was observed in both long bones and mandibular bones due to aging in PBS-injected mice, bone formation by MSM was more significant in the mandibular bones than in long bones. Since MSM is an anti-inflammatory compound, injection of MSM may have reduced the inflammatory events in the mandibular area and improved bone density. Clark et al. reported depletion of macrophages in old mice resulted in decreased inflammatory cytokines within the gingiva and reduced bone loss (Clark et al., 2021).

Furthermore, as suggested by others, it may be due to the unique characteristics of collagen in the mandible compared to the long bones (Matsuura et al., 2014). The uniqueness may include a more significant amount of collagen with a smaller amount of mature cross-links and a lower extent of Lysine hydroxylation. These structures support the mandibular matrix’s distinct interactions with bone remodeling cells, including osteoblasts, osteoclasts, and precursors (Matsuura et al., 2014). Moreover, the differentiation of osteoblasts occurs in optimal collagen cross-linking (Turecek et al., 2008). Studies have also shown that proteinases used for bone resorption in mandible displayed different properties from long bones (Azari et al., 2011; de Souza Faloni et al., 2011; Vermeer et al., 2013). Furthermore, the bone formation rate decreases with age in femoral bones, whereas it remains elevated in the jawbones (Huja and Beck, 2008). Nevertheless, irrespective of the mechanism, it is possible that the arrangement of collagen in mandibular bone may assist in bone remodeling *via* their interaction with bone remodeling cells. Therefore, the collagen matrix structure and its interaction with bone cells in the mandible may provide a notable difference in the remodeling process compared with long bones.

Studies have shown that inter-radicular bone loss is associated with the progression of bone loss in multirooted teeth in patients with chronic periodontitis (Desai and Shinde, 2012). Inter-radicular alveolar bone is exposed to occlusal stimuli and is often used for alveolar bone histomorphometry. Here, in the TRAP-stained mandibular bone sections, we have shown a significant bone loss in the inter-radicular bone region of PBS-injected mice (Figures 5A,A′); however, although the osteoclast number remains the same in both PBS- and MSM-injected mice, a considerable decrease in the bone loss was observed in MSM-injected mice (Figures 5B,B′). *In vitro* experiments with osteoclasts also demonstrated no changes in osteoclast number in MSM untreated or treated osteoclasts. We then raised the question, Is this related to an increase in bone formation?

To further determine that MSM stimulated bone formation, we analyzed the serum for bone resorption (TRAP and CTX-1) and formation (OCN and P1NP) markers. Although MSM did not affect the bone resorption of osteoclasts from RAW cells *in vitro*, a significant decrease in the bone resorption markers was observed in the serum of mice injected with MSM compared with PBS-injected mice. The levels of TRAP and CTX1 represent the measurement of enzymes and peptides released during bone resorption. As indicated by others (Milne et al., 2009), serum phosphatase levels (TRAP) can be used as an alternative measure to validate osteoclast activity. We found that MSM can reduce bone resorption. The measurement of serum levels of P1NP is precisely comparative to the amount of new collagen produced by osteoblasts. Osteocalcin level is a valid marker of bone formation and represents osteoid formation rather than mineralized bone formation. Both P1NP and OCN are currently the best and widely used indicators of bone formation (Melkko et al., 1996; Huja and Beck, 2008; Chavassieux et al., 2015). In addition to reducing bone resorption by osteoclasts, an increase in bone formation by osteoblasts may have contributed to the rise in bone density in the inter-radicular bone region of the bone.

Consistent with an increase in serum levels of OCN, immunohistochemistry analyses also displayed more OCN-positive cells in the mandibular area. The intriguing observation in the immunohistochemistry analyses is a decrease in CD105 positive cells and a corresponding increase in OCN-positive cells in the mandibular bone sections of MSM-injected mice. Several types of stem cells (DPSCs, SHED, PDLSCs, SCAPs, and DFPCs) are present in the dental tissue (Gronthos et al., 2000; Sonoyama et al., 2006, 2008; Morsczeck et al., 2009; Morsczeck and Schmalz, 2010; Morsczeck, 2015), and these stem cells can provide novel therapies in dentistry and support bone formation *in vitro* and *in vivo*. The expression of CD105 (aka endoglin) in MSC is necessary for self-renewal. These cells are shown to form bone *in vivo* and are a promising tool for bone regeneration (Aslan et al., 2006). A decrease in CD105 positive cells and a corresponding increase in OCN-positive osteoblast-like cells in MSM-injected mice strongly suggest that MSM can induce osteogenic differentiation of stem cells *in vivo*. Future studies will evaluate the mechanism and potential of CD105 positive cells to differentiate into osteoblast-like cells in the presence of MSM.

We showed in this study the initial characterization of the effects of MSM on bone formation in aging mice. Yet, we recognize that this study has limitations, and our future studies will address these limitations. More specifically, our further investigation includes analyzing the effects of MSM on: (1) bone formation using dynamic histomorphometry as shown previously (Jilka et al., 1996; Weinstein et al., 1997; Chellaiah et al., 2000) and (2) the differentiation of CD105 positive cells into OCN-positive osteoclast-like cells *in vitro* and *in vivo*. *In vitro* analysis will also focus on the molecular mechanisms by which MSM induces the osteogenic differentiation of stem cells (SHED) and increases bone formation. For example, is there any role for the Wnt pathway to stimulate the differentiation of SHED into osteoblast-like cells and bone formation?

## Conclusion

Based on histomorphometry, micro-CT, biochemical, and immunohistochemistry analyses, we have found that MSM increases bone formation in the inter-radicular region of the mandible of the aging mice. Furthermore, OCN-positive osteoblast-like cells were more in the inter-radicular areas of bone, where more bone density was observed. A decrease in CD105 positive stem cells with a concomitant increase in osteoblast-like cells suggests that MSM can induce the differentiation process *in vivo* based on the needs. Although these findings additionally support the bone remodeling effect of MSM, more studies are necessary to identify the molecular mechanisms involved in this differentiation process *in vitro* and *in vivo*. We suggest that the therapeutic effect of MSM on bone loss could go beyond alveolar bone loss that occurs in periodontitis Studies have shown that estrogen loss promotes continual inflammation, which supports post-menopausal osteoporosis (PMO). Pro-inflammatory cytokines (e.g., TNF-alpha and IL-17A) contribute to osteoclast activation and bone loss in PMO. Thus, the potent anti-inflammatory MSM can be used as a therapeutic agent to improve bone loss-associated diseases, including periodontitis, PMO, and rheumatoid arthritis.

## Data Availability Statement

The original contributions presented in the study are included in the article/Supplementary Material, further inquiries can be directed to the corresponding author.

## Ethics Statement

The experimental procedures were reviewed and approved by the Institutional Animal Care and Use Committee of the University of Maryland, Baltimore (approval number #417006, MD, United States). All experiments were performed under the relevant guidelines and regulations.

## Author Contributions

MC and HA were involved in the conceptualization, data curation, and formal analyses. HA performed the injections and maintained the animals. She also collected the tissues (blood, liver, heart, kidney, and bones) for various studies and serum to analyze biomarkers for bone resorption and bone formation by ELISA. LS and MA performed IHC staining and osteoclast studies, respectively. HA and JS conducted micro-CT scanning, data analyses, and computations. MC also completed funding acquisition, project administration, resources, supervision, validation, and writing the original draft. All authors contributed to the article and approved the submitted version.

## Author Disclaimer

Solely the authors’ responsibility and does not necessarily represent the official views of National Institutes of Health.

## Conflict of Interest

The authors declare that the research was conducted in the absence of any commercial or financial relationships that could be construed as a potential conflict of interest.

## Publisher’s Note

All claims expressed in this article are solely those of the authors and do not necessarily represent those of their affiliated organizations, or those of the publisher, the editors and the reviewers. Any product that may be evaluated in this article, or claim that may be made by its manufacturer, is not guaranteed or endorsed by the publisher.

## References

[B1] Abdelmagid S. M. Barbe M. F. Safadi F. F. (2015). Role of inflammation in the aging bones. *Life Sci.* 123 25–34. 10.1016/j.lfs.2014.11.011 25510309

[B2] Ahn H. Kim J. Lee M. J. Kim Y. J. Cho Y. W. Lee G. S. (2015). Methylsulfonylmethane inhibits NLRP3 inflammasome activation. *Cytokine* 71 223–231. 10.1016/j.cyto.2014.11.001 25461402

[B3] Aljohani H. Senbanjo L. T. Chellaiah M. A. (2019). Methylsulfonylmethane increases osteogenesis and regulates the mineralization of the matrix by transglutaminase 2 in SHED cells. *PLoS One* 14:e0225598. 10.1371/journal.pone.0225598 31805069 PMC6894810

[B4] Aljohani H. Stains J. P. Majumdar S. Srinivasan D. Senbanjo L. Chellaiah M. A. (2021). Peptidomimetic inhibitor of L-plastin reduces osteoclastic bone resorption in aging female mice. *Bone Res.* 9:22. 10.1038/s41413-020-00135-9 33837180 PMC8035201

[B5] AlQranei M. S. Aljohani H. Majumdar S. Senbanjo L. T. Chellaiah M. A. (2020). C-phycocyanin attenuates RANKL-induced osteoclastogenesis and bone resorption in vitro through inhibiting ROS levels, NFATc1 and NF-kappaB activation. *Sci. Rep.* 10:2513. 10.1038/s41598-020-59363-y 32054921 PMC7018981

[B6] Aslan H. Zilberman Y. Kandel L. Liebergall M. Oskouian R. J. Gazit D. (2006). Osteogenic differentiation of noncultured immunoisolated bone Marrow-Derived CD105+ cells. *Stem Cells* 24 1728–1737. 10.1634/stemcells.2005-0546 16601078

[B7] Azari A. Schoenmaker T. de Souza Faloni A. P. Everts V. de Vries T. J. (2011). Jaw and long bone marrow-derived osteoclasts differ in shape and their response to bone and dentin. *Biochem. Biophys. Res. Commun.* 409 205–210. 10.1016/j.bbrc.2011.04.120 21565168

[B8] Bento L. W. Zhang Z. Imai A. Nor F. Dong Z. Shi S. (2013). Endothelial differentiation of SHED requires MEK1/ERK signaling. *J. Dent. Res.* 92 51–57. 10.1177/0022034512466263 23114032 PMC3521451

[B9] Butawan M. Benjamin R. L. Bloomer R. J. (2017). Methylsulfonylmethane: applications and safety of a novel dietary supplement. *Nutrients* 9:290. 10.3390/nu9030290 28300758 PMC5372953

[B10] Cai M. Yang L. Zhang S. Liu J. Sun Y. Wang X. (2017). A bone-resorption surface-targeting nanoparticle to deliver anti-miR214 for osteoporosis therapy. *Int. J. Nanomed.* 12 7469–7482. 10.2147/IJN.S139775 29075114 PMC5648312

[B11] Chavassieux P. Portero-Muzy N. Roux J. P. Garnero P. Chapurlat R. (2015). Are biochemical markers of bone turnover representative of bone histomorphometry in 370 postmenopausal women? *J. Clin. Endocrinol. Metab.* 100 4662–4668. 10.1210/jc.2015-2957 26505821

[B12] Chellaiah M. Kizer N. Silva M. Alvarez U. Kwiatkowski D. Hruska K. A. (2000). Gelsolin deficiency blocks podosome assembly and produces increased bone mass and strength. *J. Cell Biol.* 148 665–678. 10684249 10.1083/jcb.148.4.665 PMC2169374

[B13] Chellaiah M. A. Kizer N. Biswas R. Alvarez U. Strauss-Schoenberger J. Rifas L. (2003). Osteopontin deficiency produces osteoclast dysfunction due to reduced CD44 surface expression. *Mol. Biol. Cell* 14 173–189. 12529435 10.1091/mbc.E02-06-0354 PMC140236

[B14] Clark D. Halpern B. Miclau T. Nakamura M. Kapila Y. Marcucio R. (2021). The contribution of macrophages in old mice to periodontal disease. *J. Dent. Res. [Online ahead of print]* 220345211009463. 10.1177/00220345211009463 33906501 PMC8532239

[B15] de Souza Faloni A. P. Schoenmaker T. Azari A. Katchburian E. Cerri P. S. de Vries T. J. (2011). Jaw and long bone marrows have a different osteoclastogenic potential. *Calcif Tissue Int.* 88 63–74. 10.1007/s00223-010-9418-4 20862464 PMC3021190

[B16] Demontiero O. Vidal C. Duque G. (2012). Aging and bone loss: new insights for the clinician. *Ther. Adv. Musculoskelet. Dis* 4 61–76. 10.1177/1759720X11430858 22870496 PMC3383520

[B17] Dempster D. W. Compston J. E. Drezner M. K. Glorieux F. H. Kanis J. A. Malluche H. (2013). Standardized nomenclature, symbols, and units for bone histomorphometry: a 2012 update of the report of the ASBMR Histomorphometry Nomenclature Committee. *J. Bone Miner. Res.* 28 2–17. 10.1002/jbmr.1805 23197339 PMC3672237

[B18] Desai S. Shinde H. (2012). Correlation of interdental and interradicular bone loss in patients with chronic periodontitis: a clinical and radiographic study. *Nigerian J. Clin. Practice* 15 125–131. 10.4103/1119-3077.97280 22718157

[B19] Eastell R. O’Neill T. W. Hofbauer L. C. Langdahl B. Reid I. R. Gold D. T. (2016). Post-menopausal osteoporosis. *Nat. Rev. Dis. Primers* 2:16069. 10.1038/nrdp.2016.69 27681935

[B20] Gibon E. Lu L. Y. Nathan K. Goodman S. B. (2017). Inflammation, ageing, and bone regeneration. *J. Orthop. Translat.* 10 28–35. 10.1016/j.jot.2017.04.002 29094003 PMC5662134

[B21] Goldring S. R. (2003). Inflammatory mediators as essential elements in bone remodeling. *Calcif Tissue Int.* 73 97–100. 10.1007/s00223-002-1049-y 14565589

[B22] Gregory P. J. Sperry M. Wilson A. F. (2008). Dietary supplements for osteoarthritis. *Am. Fam. Physician* 77 177–184. 18246887

[B23] Gronthos S. Mankani M. Brahim J. Robey P. G. Shi S. (2000). Postnatal human dental pulp stem cells (DPSCs) in vitro and in vivo. *Proc. Natl. Acad. Sci. U.S.A.* 97 13625–13630. 10.1073/pnas.240309797 11087820 PMC17626

[B24] Gupta A. Cao W. Chellaiah M. A. (2012). Integrin avb3 and CD44 pathways in metastatic prostate cancer cells support osteoclastogenesis via RUNX2/Smad5/RANKL signaling axis. *Mol. Cancer* 11:66. 10.1186/1476-4598-11-66 22966907 PMC3499378

[B25] Ha S. H. Choung P. H. (2020). MSM promotes human periodontal ligament stem cells differentiation to osteoblast and bone regeneration. *Biochem. Biophys. Res. Commun.* 528 160–167. 10.1016/j.bbrc.2020.05.097 32466845

[B26] Haffajee A. D. Socransky S. S. Lindhe J. Kent R. L. Okamoto H. Yoneyama T. (1991). Clinical risk indicators for periodontal attachment loss. *J. Clin. Periodontol.* 18 117–125. 10.1111/j.1600-051x.1991.tb01700.x 2005225

[B27] Huja S. S. Beck F. M. (2008). Bone remodeling in maxilla, mandible, and femur of young dogs. *Anat Rec. (Hoboken)* 291 1–5. 10.1002/ar.20619 18085627

[B28] Huttner E. A. Machado D. C. Oliveira R. B. Antunes A. G. F. Hebling E. (2009). Effects of human aging on periodontal tissues. *Spec. Care Dent.* 29 149–155. 10.1111/j.1754-4505.2009.00082.x 19573041

[B29] Ismail A. I. Morrison E. C. Burt B. A. Caffesse R. G. Kavanagh M. T. (1990). Natural history of periodontal disease in adults: findings from the Tecumseh Periodontal Disease Study, 1959-87. *J. Dent. Res.* 69 430–435. 10.1177/00220345900690020201 2407756

[B30] Jilka R. L. (2013). The relevance of mouse models for investigating age-related bone loss in humans. *J. Gerontol. A Biol. Sci. Med. Sci.* 68 1209–1217. 10.1093/gerona/glt046 23689830 PMC3779631

[B31] Jilka R. L. Weinstein R. S. Takahashi K. Parfitt A. M. Manolagas S. C. (1996). Linkage of decreased bone mass with impaired osteoblastogenesis in a murine model of accelerated senescence. *J. Clin. Invest* 97 1732–1740. 8601639 10.1172/JCI118600 PMC507238

[B32] Jonasson G. Rythen M. (2016). Alveolar bone loss in osteoporosis: a loaded and cellular affair? *Clin. Cosmet Investig. Dent.* 8 95–103. 10.2147/CCIDE.S92774 27471408 PMC4948717

[B33] Joung Y. H. Lim E. J. Darvin P. Chung S. C. Jang J. W. Do P. K. (2012). MSM enhances GH signaling via the Jak2/STAT5b pathway in osteoblast-like cells and osteoblast differentiation through the activation of STAT5b in MSCs. *PLoS. One* 7:e47477. 10.1371/journal.pone.0047477 23071812 PMC3469535

[B34] Khosla S. Oursler M. J. Monroe D. G. (2012). Estrogen and the skeleton. *Trends Endocrinol. Metab.* 23 576–581. 10.1016/j.tem.2012.03.008 22595550 PMC3424385

[B35] Kim L. S. Axelrod L. J. Howard P. Buratovich N. Waters R. F. (2006). Efficacy of methylsulfonylmethane (MSM) in osteoarthritis pain of the knee: a pilot clinical trial. *Osteoarthritis Cartilage* 14 286–294. 10.1016/j.joca.2005.10.003 16309928

[B36] Kim Y. H. Kim D. H. Lim H. Baek D. Y. Shin H. K. Kim J. K. (2009). The anti-inflammatory effects of methylsulfonylmethane on lipopolysaccharide-induced inflammatory responses in murine macrophages. *Biol. Pharm. Bull.* 32 651–656. 10.1248/bpb.32.651 19336900

[B37] Koduganti R. R. Gorthi C. Reddy P. V. Sandeep N. (2009). Osteoporosis: “A risk factor for periodontitis”. *J. Ind. Soc. Periodontol.* 13 90–96. 10.4103/0972-124X.55841 PMC2847131 20407657

[B38] Liang S. Hosur K. B. Domon H. Hajishengallis G. (2010). Periodontal inflammation and bone loss in aged mice. *J. Periodontal. Res.* 45 574–578. 10.1111/j.1600-0765.2009.01245.x 20337897 PMC2894296

[B39] Lubis A. M. T. Siagian C. Wonggokusuma E. Marsetyo A. F. Setyohadi B. (2017). Comparison of glucosamine-chondroitin sulfate with and without methylsulfonylmethane in grade I-II knee osteoarthritis: a double blind randomized controlled trial. *Acta Med. Indones* 49 105–111. 28790224

[B40] Ma T. Sadashivaiah K. Chellaiah M. A. (2010). Regulation of sealing ring formation by L-plastin and cortactin in osteoclasts. *J. Biol. Chem* 285 29911–29924. 20650888 10.1074/jbc.M109.099697 PMC2943304

[B41] Matsuura T. Tokutomi K. Sasaki M. Katafuchi M. Mizumachi E. Sato H. (2014). Distinct characteristics of mandibular bone collagen relative to long bone collagen: relevance to clinical dentistry. *BioMed. Res. Int.* 2014:769414. 10.1155/2014/769414 24818151 PMC4004038

[B42] Melkko J. Kauppila S. Niemi S. Risteli L. Haukipuro K. Jukkola A. (1996). Immunoassay for intact amino-terminal propeptide of human type I procollagen. *Clin. Chem.* 42(Pt 1) 947–954. 8665688

[B43] Milne T. J. Ichim I. Patel B. McNaughton A. Meikle M. C. (2009). Induction of osteopenia during experimental tooth movement in the rat: alveolar bone remodelling and the mechanostat theory. *Eur. J. Orthod.* 31 221–231. 10.1093/ejo/cjp032 19458288

[B44] Moorer M. C. Hebert C. Tomlinson R. E. Iyer S. R. Chason M. Stains J. P. (2017). Defective signaling, osteoblastogenesis and bone remodeling in a mouse model of connexin 43 C-terminal truncation. *J. Cell Sci.* 130 531–540. 10.1242/jcs.197285 28049723 PMC5312734

[B45] Morsczeck C. (2015). Molecular mechanisms in dental follicle precursor cells during the osteogenic differentiation. *Histol. Histopathol.* 30 1161–1169. 10.14670/HH-11-634 26016694

[B46] Morsczeck C. Frerich B. Driemel O. (2009). Dental stem cell patents. *Recent Pat. DNA Gene Seq.* 3 39–43. 10.2174/187221509787236200 19149737

[B47] Morsczeck C. Schmalz G. (2010). Transcriptomes and proteomes of dental follicle cells. *J. Dent. Res.* 89 445–456. 10.1177/0022034510366899 20348482

[B48] Mundy G. R. (2007). Osteoporosis and inflammation. *Nutr. Rev.* 65(Pt 2) S147–S151. 18240539 10.1111/j.1753-4887.2007.tb00353.x

[B49] Pacifici R. (2008). Estrogen deficiency, T cells and bone loss. *Cell Immunol* 252 68–80. 17888417 10.1016/j.cellimm.2007.06.008

[B50] Papapanou P. N. Wennstrom J. L. Grondahl K. (1989). A 10-year retrospective study of periodontal disease progression. *J. Clin. Periodontol.* 16 403–411. 10.1111/j.1600-051x.1989.tb01668.x 2768535

[B51] Sonoyama W. Liu Y. Fang D. Yamaza T. Seo B. M. Zhang C. (2006). Mesenchymal stem cell-mediated functional tooth regeneration in swine. *PLoS One* 1:e79. 10.1371/journal.pone.0000079 17183711 PMC1762318

[B52] Sonoyama W. Liu Y. Yamaza T. Tuan R. S. Wang S. Shi S. (2008). Characterization of the apical papilla and its residing stem cells from human immature permanent teeth: a pilot study. *J. Endod.* 34 166–171. 10.1016/j.joen.2007.11.021 18215674 PMC2714367

[B53] Soysa N. S. Alles N. (2016). Osteoclast function and bone-resorbing activity: an overview. *Biochem. Biophys. Res. Commun* 476 115–120. 10.1016/j.bbrc.2016.05.019 27157135

[B54] Turecek C. Fratzl-Zelman N. Rumpler M. Buchinger B. Spitzer S. Zoehrer R. (2008). Collagen cross-linking influences osteoblastic differentiation. *Calcif Tissue Int.* 82 392–400. 10.1007/s00223-008-9136-3 18488133

[B55] Usha P. R. Naidu M. U. (2004). Randomised, double-blind, parallel, placebo-controlled study of oral glucosamine, methylsulfonylmethane and their combination in osteoarthritis. *Clin. Drug Investig.* 24 353–363. 10.2165/00044011-200424060-00005 17516722

[B56] Vermeer J. A. Jansen I. D. Marthi M. Coxon F. P. McKenna C. E. Sun S. (2013). Jaw bone marrow-derived osteoclast precursors internalize more bisphosphonate than long-bone marrow precursors. *Bone* 57 242–251. 10.1016/j.bone.2013.08.007 23962725

[B57] Weinstein R. S. Jilka R. L. Parfitt A. M. Manolagas S. C. (1997). The effects of androgen deficiency on murine bone remodeling and bone mineral density are mediated via cells of the osteoblastic lineage. *Endocrinology* 138 4013–4021. 9275093 10.1210/endo.138.9.5359

[B58] Weitzmann M. N. Pacifici R. (2006). Estrogen deficiency and bone loss: an inflammatory tale. *J. Clin. Invest.* 116 1186–1194. 16670759 10.1172/JCI28550 PMC1451218

[B59] Willinghamm M. D. Brodt M. D. Lee K. L. Stephens A. L. Ye J. Silva M. J. (2010). Age-related changes in bone structure and strength in female and male BALB/c mice. *Calcif. Tissue Int.* 86 470–483. 10.1007/s00223-010-9359-y 20405109 PMC2895262

