# Supplementary material for: Methylsulfonylmethane Increases the Alveolar Bone Density of Mandibles in Aging Female Mice
Source: Front Physiol. 2021 Oct 4;12:708905. doi: 10.3389/fphys.2021.708905 (PMC8521043; doi:10.3389/fphys.2021.708905)
Supplement: Supplementary file 1 [file Data_Sheet_1.pdf]

Supplementary Figures

Methylsulfonylmethane increases the alveolar bone density of mandibles in aging female mice

Hanan Aljohani[1,2], Linda Senbanjo[1], Mohammed S AlQranei[1,3], Joseph P. Stains[4], Meenakshi A. Chellaiah[1*]

[1] Department of Oncology and Diagnostic Sciences, School of Dentistry, University of Maryland, Baltimore, Maryland, USA

[2]Department of Oral Medicine and Diagnostics Sciences, King Saud University, School of Dentistry, Riyadh, KSA

[3] Preventive Dental Sciences Department, School of Dentistry, Imam Abdulrahman Bin Faisal University, Dammam, Saudi Arabia.

[4] Department of Orthopedics, University of Maryland School of Medicine, Baltimore, MD, USA

*** Correspondence:**
Meenakshi A. Chellaiah
mchellaiah@umaryland.edu

Supplementary Figure 1

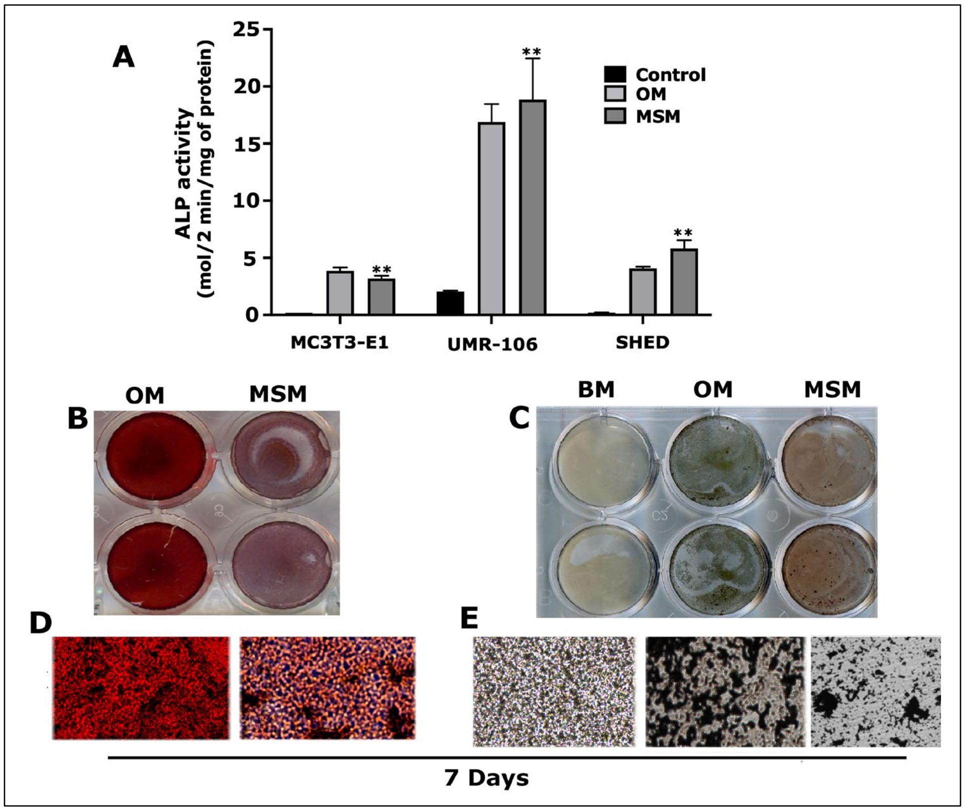

Figure S1: MSM effect on Alkaline phosphatase enzyme activity in different cell lines and mineralization in UMR 106 cells. A. The alkaline phosphatase enzyme activity in MC3T3, UMR-106, and SHED cells. These cells were incubated with osteogenic medium (OM) and MSM for 7 days (16). Corresponding cells cultured in the basal medium (BM) were used as controls. Alkaline phosphatase enzyme activity (A) and mineralization assays (B and C) were done as described previously (16). Data are expressed as mean ± SEM (n=3), and statistical analysis was done using a one-way ANOVA test; $p<0.001$ in MSM treated cells vs. cells incubated in BM. Mineralized matrix formation was analyzed by Alizarine Red Staining (ARS; panels B and D) and von Kossa (VK.; panels C and E) staining after incubation of UMR cells with OM and MSM for 7 days. UMR cells cultured in basal medium (BM) are shown in VK staining. Representative magnified phase-contrast micrographs are shown in D and E (magnification is 40X). Data showed representative of three independent experiments.

Supplementary Figure 2

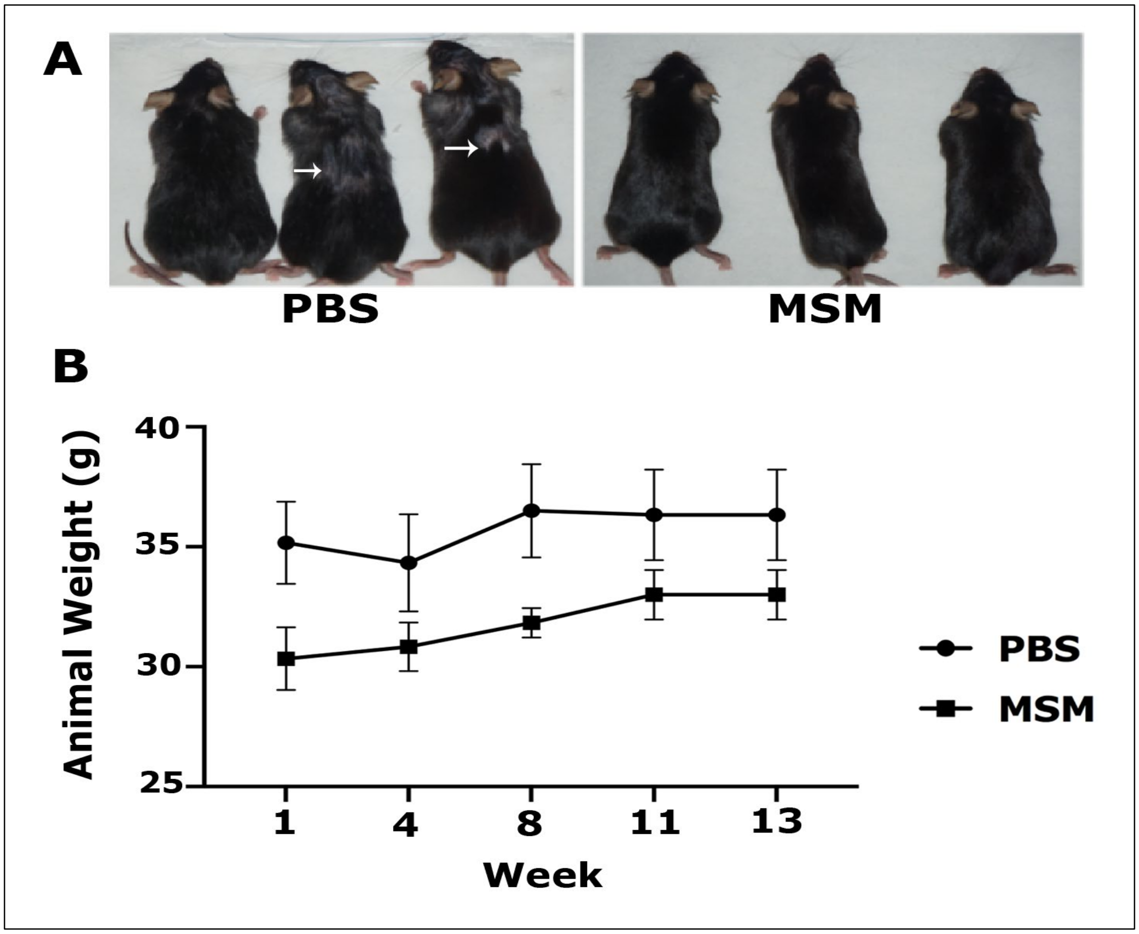

Figure S2: General analyses in mice injected with PBS and MSM for 13 weeks. Six aging mice at the age of 36 were injected with PBS and MSM for 13 weeks, and three from each group photographed on week 13 are shown. **(A)**. All mice from each group were weighed every 4 weeks until 8 weeks and every other week until 13 weeks. Bodyweight data are provided as mean ±SEM in a line graph **(B)**. Statistically, there was no significant change in the body weight between the two groups tested.

Supplementary Figure 3

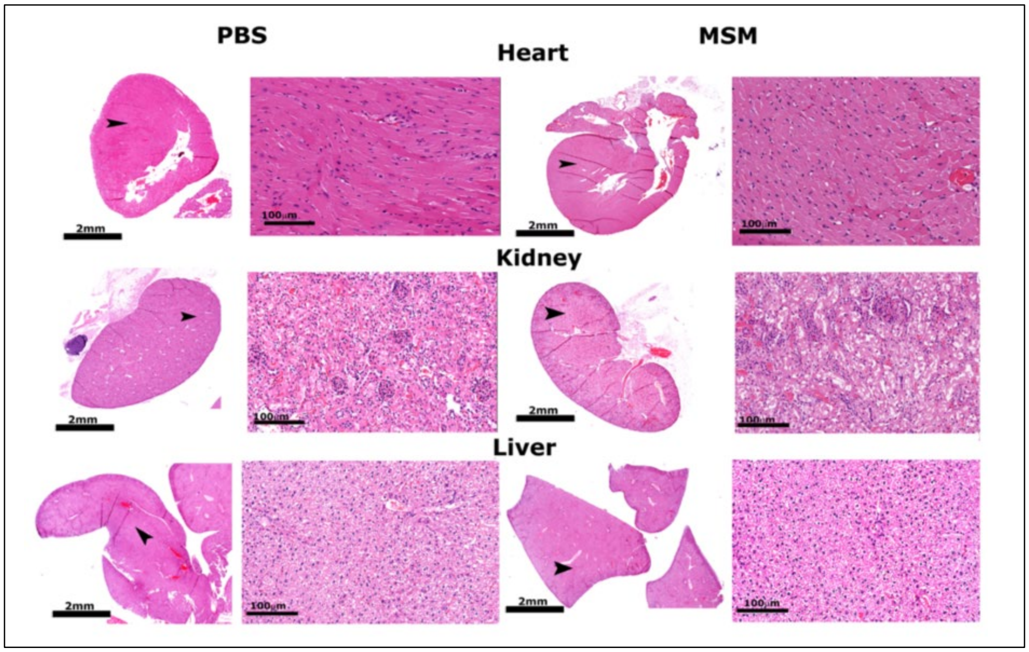

Figure S3 Histological sections of the organs (Heart, Kidney, Liver) isolated from mice injected with PBS and MSM.

Organs were harvested from three mice and subjected to H&E staining to detect any abnormalities. A representative histological section of the soft organs from each injection is shown. An arrowhead in each organ section in the left panel indicates the enlarged area, as shown in the right panel. Scale bar: 2 mm (left panels) and 100μm (right panels).

Supplementary Figure 4

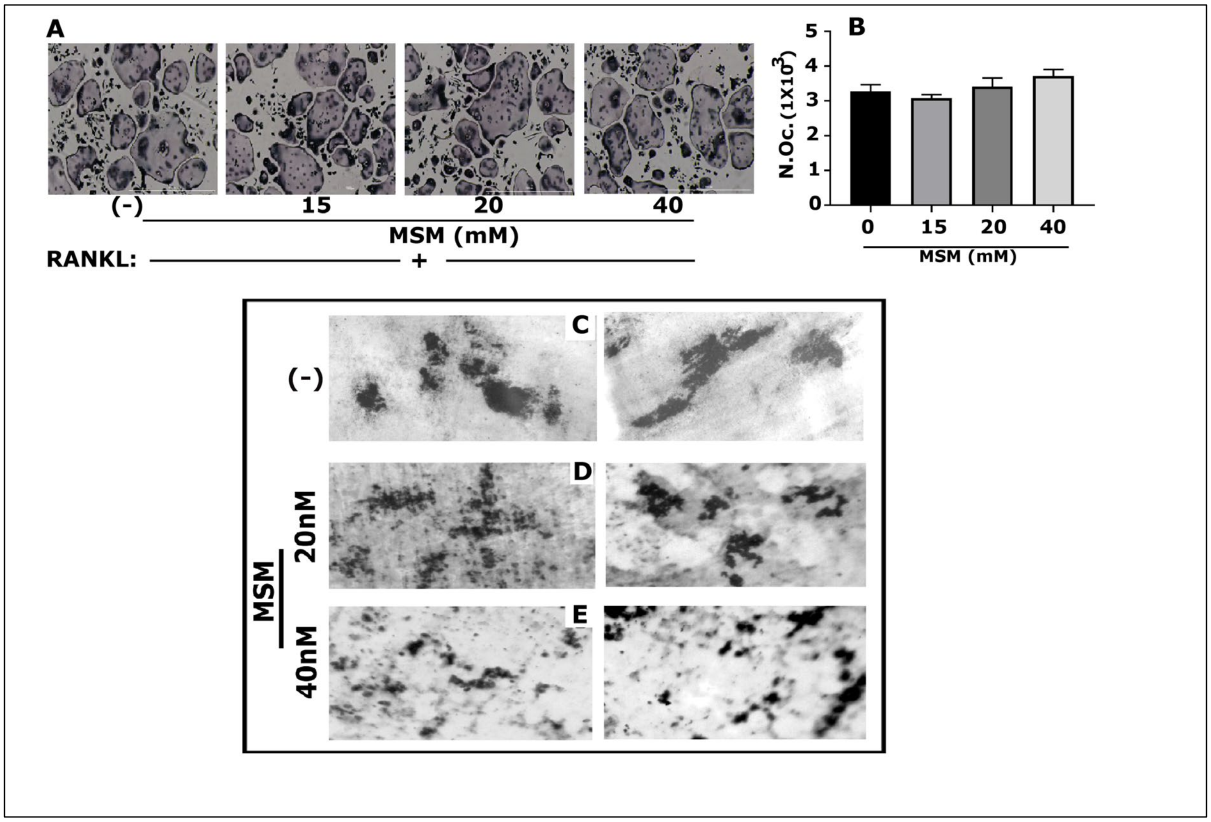

Figure S4 Analysis of the effect of MSM on RANKL-mediated osteoclast differentiation and dentine matrix resorption.

The effect of different concentrations of MSM (15 mM, 20 mM, and 40 mM) on osteoclast differentiation **(A and B)** and dentin resorption **(C)** in the presence of RANKL is shown. Untreated (-) cells with MSM were used as controls. Procedures for treating osteoclasts with MSM and bone resorption assays are provided under osteoclast studies in the Methods section. **(A)** Phase-contrast images of TRAP-stained osteoclasts. Magnification is X40. **(B)** Each treatment was performed in triplicates. The number of TRAP-positive multinucleated osteoclasts at each concentration in triplicate was counted and plotted. Statistical analysis was performed using one-way ANOVA. No

statistically significant differences were found between the groups. Data represent the mean ± SEM of one experiment. **(C-E)** A Representative phase-contrast image of the resorption area for the indicated treatments is shown. Magnification is X100. The data showed (A-E) are representative of three independent experiments.
